# Supplementary material for: Aphrodisiac Performance of Bioactive Compounds from Mimosa pudica Linn.: In Silico Molecular Docking and Dynamics Simulation Approach
Source: Molecules. 2022 Jun 13;27(12):3799. doi: 10.3390/molecules27123799 (PMC9229059; doi:10.3390/molecules27123799)
Supplement: Supplementary file 1 [file molecules-27-03799-s001.zip › molecules-1735970-supplementary.pdf]

Table S1. Bioactive molecules from *Mimosa pudica* Linn.

S. No	Compound ID	Bioactive Molecules	Structure	Literature source
1.	94477	Mimosinamine	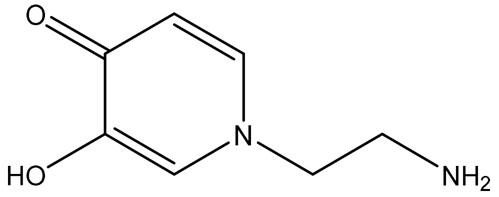	[72]
2.	125409	Beta-D-xylopyranose	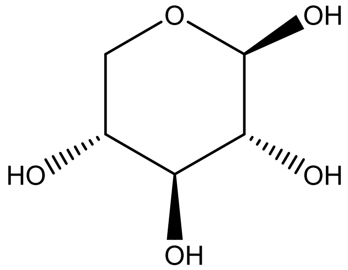	[34]
3.	190359	Mimosinic Acid	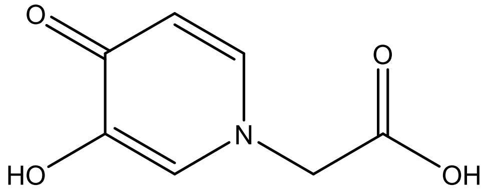	[72]
4.	370	Gallic Acid	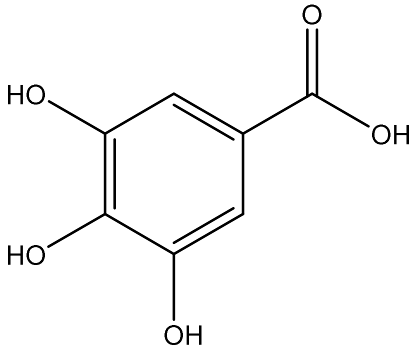	[34]
5.	951	Dl-Norepinephrine	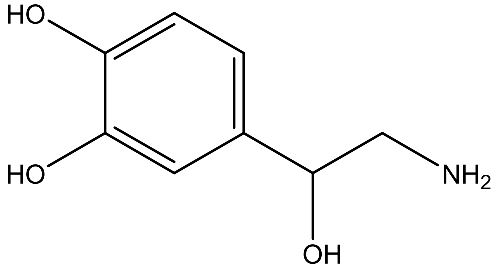	[34]

6.	164619	D-Pinitol	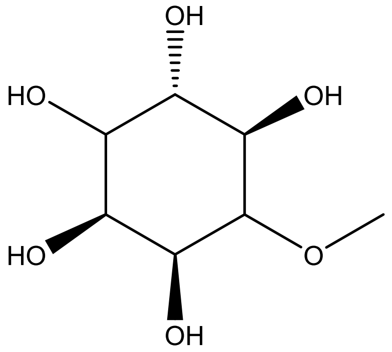	[73]
7.	3862	Mimosine	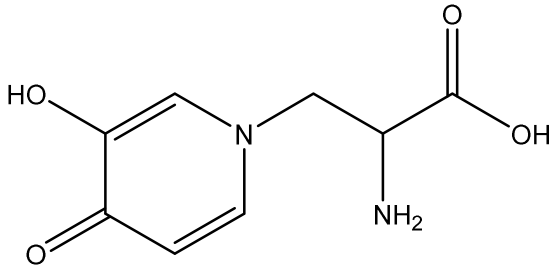	[34,73]
8.	1153	DL-tyrosine	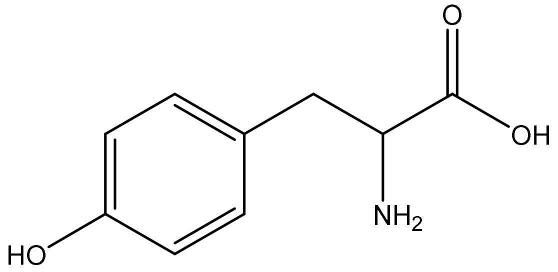	[34]
9.	71684438	Octadecadienoicacid	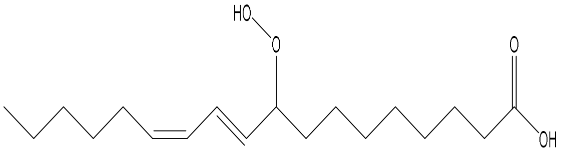	[34]
10.	94715	D-Glucopyranuronate	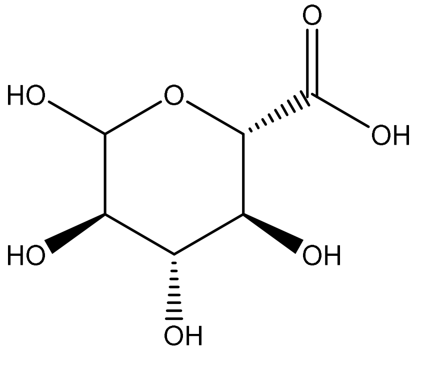	[72]

11.	5280441	Vitexin	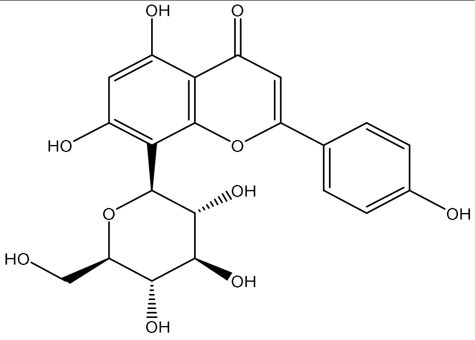	[74]
12.	440473	L-Mimosine	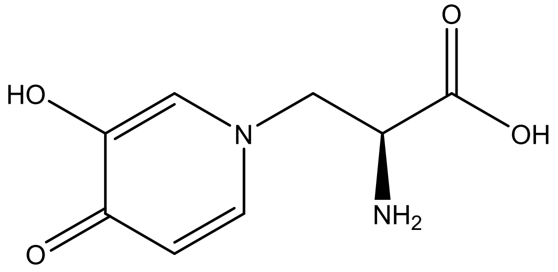	[75]
13.	5281166	Jasmonic Acid	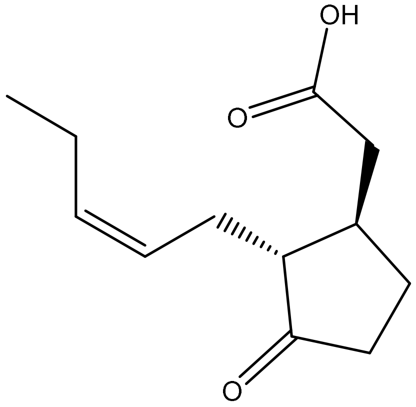	[76]
14.	5375199	Abscisic acid	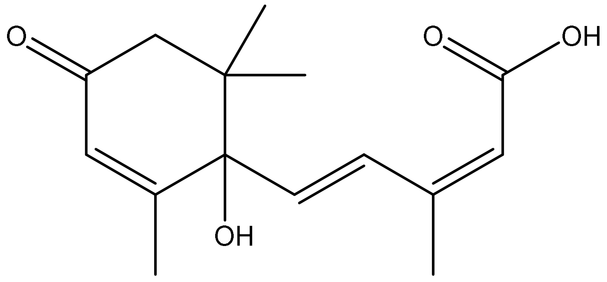	[73]
15.	100927206	Mimopudine	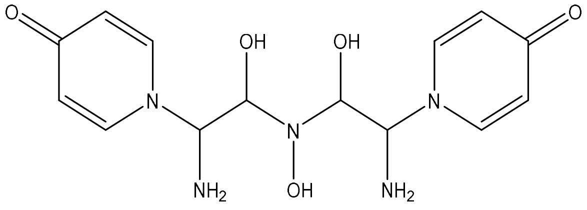	[75]

16.	5280489	Beta-Carotene	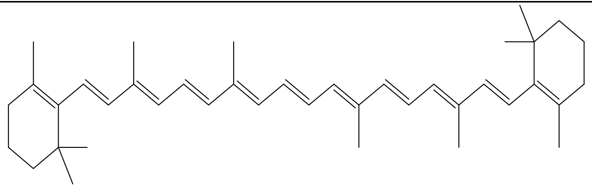	[34]
17.	5281679	Methylquercetin	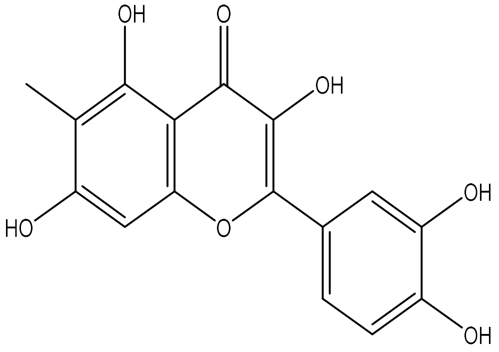	[73]
18.	64971	Betulinic Acid	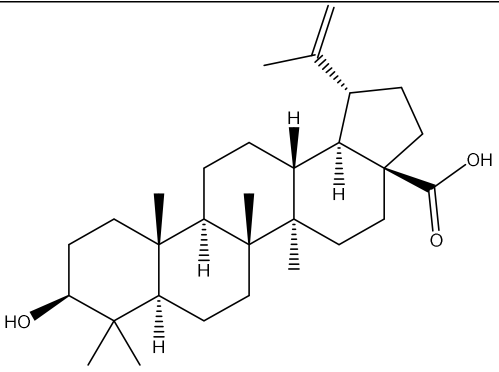	[73]
19.	222284	Beta-sitosterol	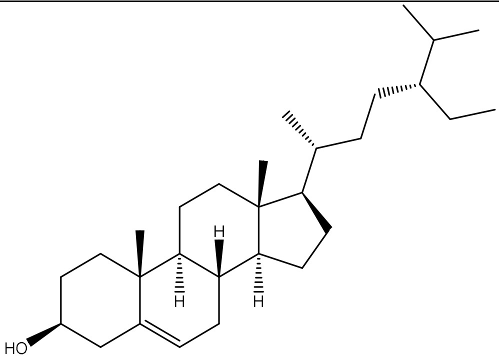	[77]

20.	5490064	Avicularin	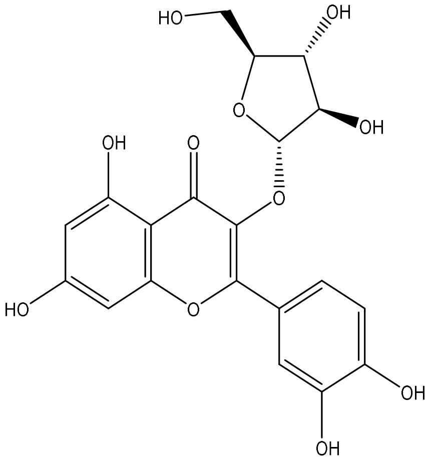	[78]
21.	5281675	Orientin	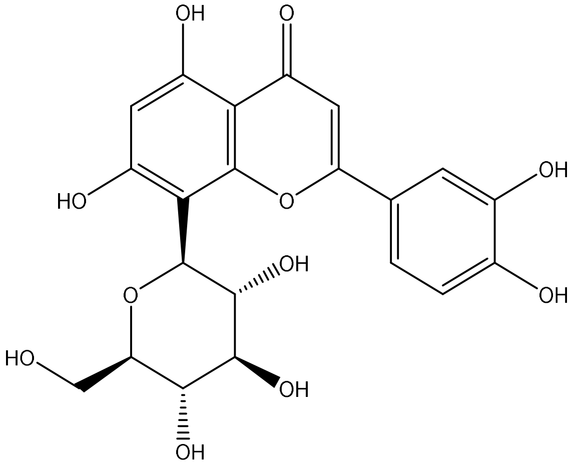	[72]
22.	114776	Isoorientin	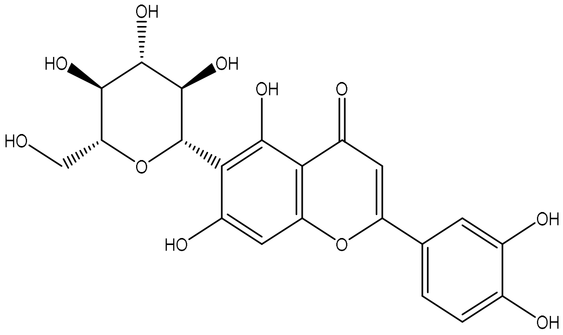	[72]
23.	70698280	Cassiaoccidentalin B	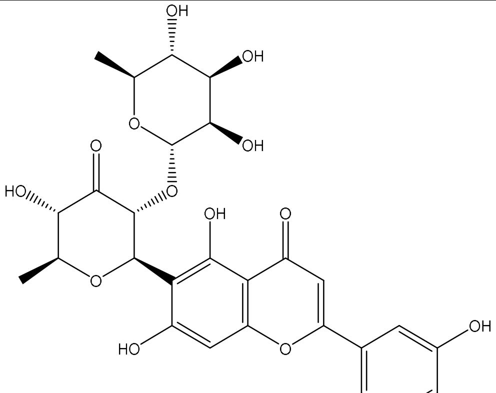	[72]

24.	5280804	Isoquercitrin	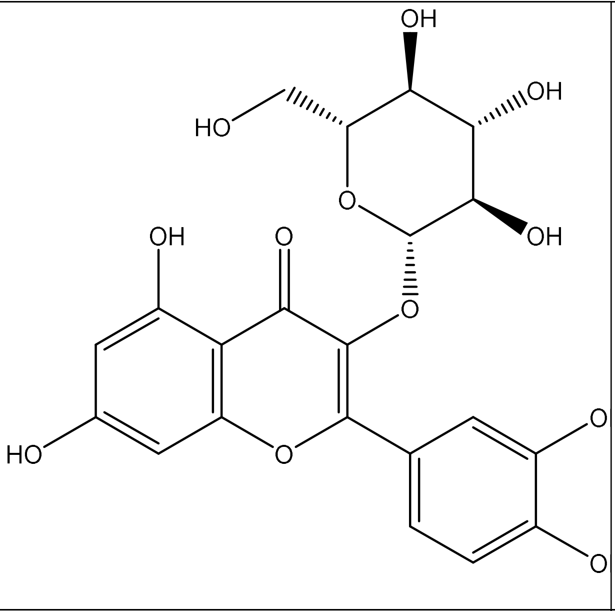	[72]
25.	162350	Isovitexin	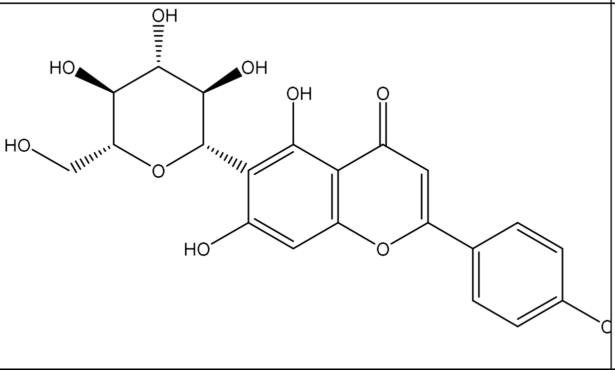	[74]
26.	5280704	Apigetrin	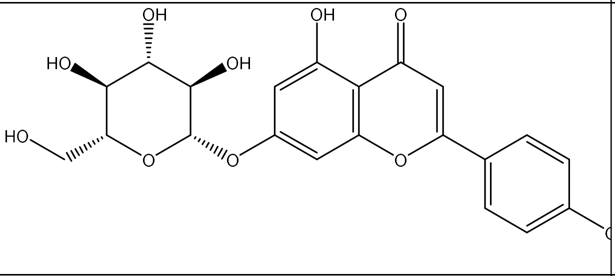	[73]
27.	5280794	Stigmasterol	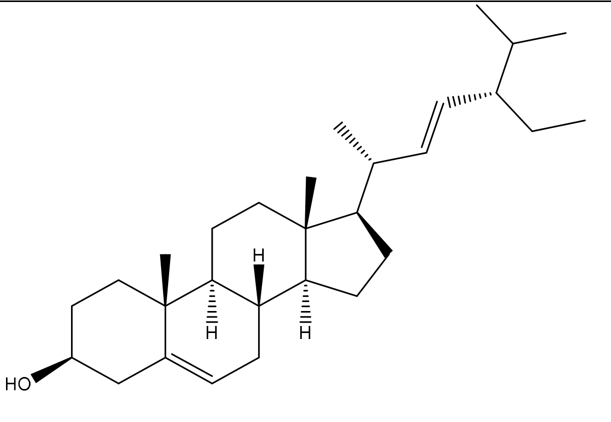	[74]

28.	46173848	Bufadienolide	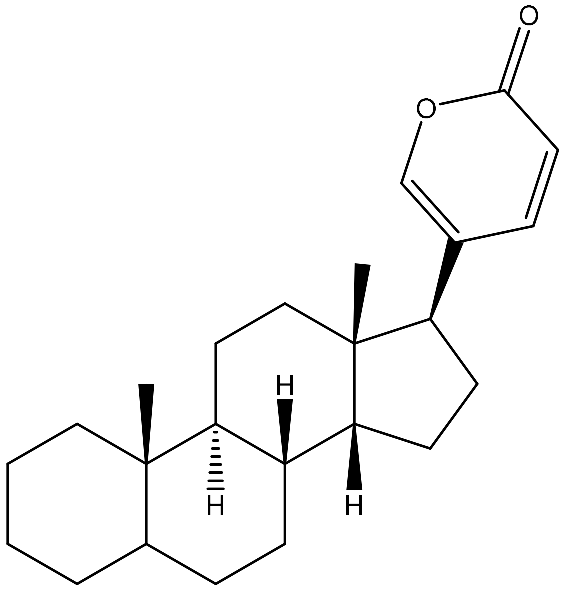	[72]

Table S2. Details of bonding interactions between phosphodiesterase type 5 enzyme with selected bioactive molecules and standard drug.

Compounds	Residues	Amino acid	Distance (Å)	Bond category
Bufadienolide	612A	TYR	3.53	Hydrophobic
	662A	ASN	3.77	Hydrophobic
	725A	LEU	3.48	Hydrophobic
	725A	LEU	3.72	Hydrophobic
	782A	VAL	3.29	Hydrophobic
	786A	PHE	3.39	Hydrophobic
	820A	PHE	3.45	Hydrophobic
	820A	PHE	3.67	Hydrophobic
	820A	PHE	3.7	Hydrophobic
	775A	GLN	2.23	Hydrogen
	817A	GLN	2.97	Hydrogen
Stigmasterol	725A	LEU	3.09	Hydrophobic
	765A	LEU	3.74	Hydrophobic
	765A	LEU	3.58	Hydrophobic
	767A	ALA	3.7	Hydrophobic
	768A	ILE	3.43	Hydrophobic
	778A	ILE	3.97	Hydrophobic
	782A	VAL	3.4	Hydrophobic
	786A	PHE	3.62	Hydrophobic
	820A	PHE	3.69	Hydrophobic
	724A	ASP	2.19	Hydrogen

	724A	ASP	2.03	Hydrogen
	725A	LEU	3.23	Hydrogen
Apigetrin	725A	LEU	3.85	Hydrophobic
	820A	PHE	3.66	Hydrophobic
	820A	PHE	3.86	Hydrophobic
	612A	TYR	1.77	Hydrogen
	612A	TYR	2.18	Hydrogen
	613A	HIS	2.63	Hydrogen
	654A	ASP	3.03	Hydrogen
	657A	HIS	3.12	Hydrogen
	662A	ASN	2.61	Hydrogen
	682A	GLU	2.94	Hydrogen
	685A	HIS	2.09	Hydrogen
	724A	ASP	2.61	Hydrogen
	786A	PHE	5.46	π-Stacking
	820A	PHE	4.04	π-Stacking
	613A	HIS	5.49	π-Cation Interactions
Isovitexin	765A	LEU	3.87	Hydrophobic
	767A	ALA	3.95	Hydrophobic
	768A	ILE	3.79	Hydrophobic
	782A	VAL	3.69	Hydrophobic
	612A	TYR	2.88	Hydrogen
	613A	HIS	3.58	Hydrogen
	661A	ASN	1.91	Hydrogen
	662A	ASN	3.52	Hydrogen
	662A	ASN	3.06	Hydrogen
	723A	THR	2.54	Hydrogen
	724A	ASP	2.03	Hydrogen
	725A	LEU	1.8	Hydrogen
	764A	ASP	2.16	Hydrogen
	767A	ALA	3.09	Hydrogen
	775A	GLN	2.06	Hydrogen
Sildenafil	612A	TYR	3.59	Hydrophobic
	813A	ILE	3.74	Hydrophobic
	817A	GLN	3.83	Hydrophobic
	820A	PHE	3.91	Hydrophobic
	613A	HIS	2.22	Hydrogen
	786A	PHE	5.34	π-Stacking

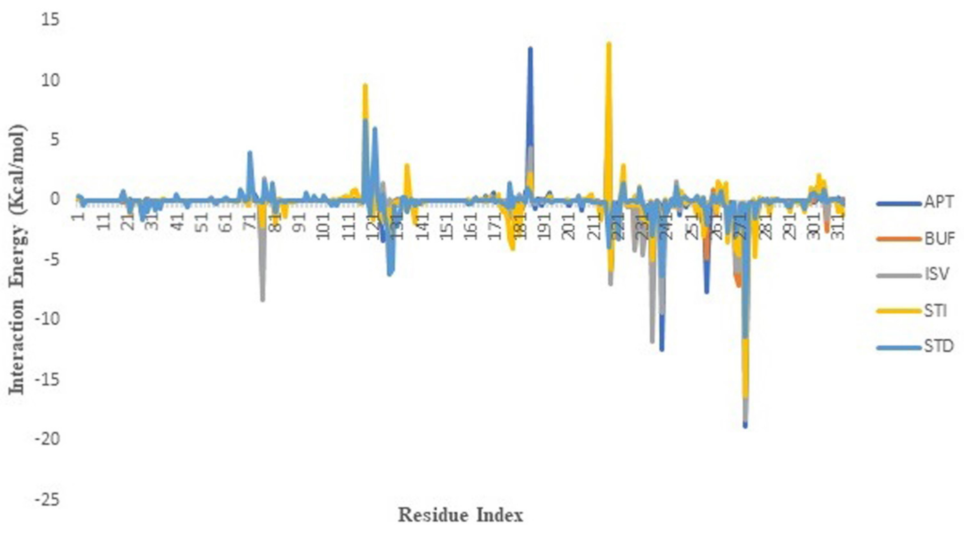

Figure S1. Per-residue energy decomposition of PDE5-APO (Black), PDE5-BUF (Green), PDE5-STI (Yellow), PDE5-ISV (Blue), PDE5-APT (Red) and PDE5-STD drug Sildenafil (Brown).
